# Estimation of Genetic and Phenotypic Parameters for Production Traits and Somatic Cell Count for Jersey Dairy Cattle in Zimbabwe

**DOI:** 10.1155/2013/470585

**Published:** 2013-07-11

**Authors:** Edward Missanjo, Venancio Imbayarwo-Chikosi, Tinyiko Halimani

**Affiliations:** ^1^Malawi College of Forestry and Wildlife, Private Bag 6, Dedza, Malawi; ^2^Department of Animal Science, Faculty of Agriculture, University of Zimbabwe, P.O. Box MP 167, Mount Pleasant, Harare, Zimbabwe

## Abstract

Genetic and phenotypic parameters for production traits and somatic cell count (SCC) for Jersey dairy cattle in Zimbabwe were estimated. A total of 10986 lactation records were obtained from Zimbabwe Livestock Identification Trust, with cows calving in the period from 1996 to 2008. An ASReml program fitting an animal model was used for the analyses. Heritability estimates for milk yield, fat yield, protein yield, fat percentage, protein percentage, and Log_10_SCC were 0.30, 0.32, 0.33, 0.42, 0.44, and 0.08, respectively. The corresponding repeatability estimates were 0.39, 0.38, 0.39, 0.49, 0.51, and 0.16, respectively. The genetic and phenotypic correlations between different production traits ranged from −0.86 to 0.95 and from −0.88 to 0.98, respectively. The genetic and phenotypic correlations between production traits and Log_10_SCC were weak almost nonsignificantly differentl from zero. The results imply that milk traits for Jersey dairy cattle in Zimbabwe are more heritable. Therefore, these traits may be preferred by breeders as selection criteria for development of effective genetic improvement programme.

## 1. Introduction

Knowledge of genetic parameters is the basis of sound livestock improvement programmes. Estimates of heritabilities and genetic correlations are essential population parameters required in animal breeding research and in design and application of practical animal breeding programmes. Genetic parameters are a characteristic of the population in which they were estimated and may change overtime due to selection and management decisions [1].

The Jersey is one of the dairy breeds found in Zimbabwe. It is the second most important breed of dairy after Holstein-Friesian. It is a small breed of dairy cattle, with mature live weight ranging from 360 to 540 kg. Originally bred on the British Channel Island of Jersey, the breed is popular for the high butterfat content of its milk and the low maintenance costs incurred due to its lower bodyweight, as well as its genial disposition. In addition, Jerseys can thrive on locally produced food. They can tolerate high temperatures; heifers mature more quickly than those of other breeds and can be mated at 13 to 15 months; they produce an average herd milk production of 3500 to 5000 kg [2].

Jersey is an ideal breed for crossbreeding with *Bos indicus* to produce a hardy, disease-tolerant, dairy-type cow which does not need a high plane of nutrition to produce reasonable milk yield and is suited to dairying in the communal areas [1]. However, there has been a rapid decrease in average milk yield per cow for Zimbabwean Jersey from 5000 kg to 3900 kg from the year 2000 to 2010 [3]. Furthermore, there is paucity of information on production traits of Jersey cattle in Zimbabwe as most researches have been concentrated on Holstein-Friesian breed [4]. In addition, there have been massive changes in the dairy farming industry since 2000 due to the land reform programme. This may have resulted in the conversion of use of some dairy farms into crop agriculture; a subsequent decrease in herd sizes; and changes to a number of management and production parameters within the herds. Imports of semen and replacement stock were also affected. This may have altered variance components and hence the genetic parameters for yield in most dairy herds including the Jersey herds. 

It thus becomes imperative to genetically evaluate the Jersey production traits as a way of increasing productivity through selection. The objective of this study was to analyse lactation records in order to fit an appropriate model for evaluation of the Zimbabwean Jersey dairy cattle regarding production traits and somatic cell count (SCC). Genetic and phenotypic parameters were also estimated. This led to the development of a selection index [5].

## 2. Materials and Methods 

### 2.1. Environment

Zimbabwe is located in Southern Africa in the tropical savannah region. The total land area is 390,759 km^2^, and it is divided into five agroecological regions. Rainfall patterns and crop production progressively deteriorate from region I to V. However, livestock production including dairying is practised in all the regions. In the regions with low rainfall, dairying is assisted by production of drought-resistant fodder crops. Most dairy farms are located within 40 km of the major cities and towns [6]. 

## 3. Data and Data Edits

The standard 305-day milk production records of pure bred Jersey were obtained from Zimbabwe Livestock Identification Trust (LIT). The file contained production records of 25 herds, with cows calving during the period 1996 to 2008. The following production traits were recorded: 305-day milk, fat and protein yields; milk yield over the complete lactation period; length of lactation; calving interval; days dry; days open and somatic cell count. Identity of herd, cow, dam, sire, date of birth, and date of calving were also recorded. 

The records were edited using Statistical Analysis of Systems version 9.1.3 [7] and Pedigree Viewer version 6.4 b [8]. Edits included removal of herds with no information on fat, protein, or milk yields and records where calving date, days dry, days open, date of birth, somatic cell count, or length of lactation had not been recorded. Calving interval classes of 20-day interval were formed from 300 to 460 days. The first class had a calving interval of zero, and this represented the first lactation cows only. Calving intervals below 300 days were deleted and those above 460 days were pooled. Further, those with 305-day milk yield less than 3000 kg and greater than 6000 kg were deleted. Days dry classes of 15-day interval from 0 to 150 days were also formed. Dry periods greater than 150 days were pooled. Age at calving was classified within parity as shown in Table 1. All duplicate records were deleted in order to remove bias from the data. Further edits imposed a restriction of at least five daughters per sire. This was done to improve estimation of the sire's breeding values and the accuracy of the estimated heritability since the biggest influence on the accuracy of estimation of heritability is the number of daughters per sire [4]. The restriction also ensured connectedness in the data and reduced bias due to preferential treatment of daughters of a particular sire in a group [1]. Then, four subdata sets were created. Data set 1 was for all lactations. This was for those records of cows with at least two consecutive lactations. Data sets 2 and 3 were for those records of cows in lactations 1 and 2, respectively. Data set 4 was for those records of cows in lactation 3 and above. The characteristics of the data sets for the traits analysed are given in Tables 2 and 3.

## 4. Statistical Analysis

Multivariate analyses to estimate variance components, heritability, predicted breeding values (EBVs), and genetic and phenotypic corrections were done using animal models and the ASReml program developed by [9]. 

Two models were fitted to the data. Model 1 (a repeatability animal model) was for data sets 1 and 4. The model is described as follows:

(1) Yijkl=μ+HYSi+CIj+DDk+b1AC⁡+b2AC⁡+al+cl+eijkl,

where  Y_*ijkl*_ is the observed value for all traits (305-day milk yield; fat and protein yields); *μ* is the overall mean common to all observations; HYS_*i*_ is the fixed effect of herd year of calving-season of calving *i*  (*i* = 1,2, 3,…, *n*); CI_*j*_ is the fixed effect of classes of calving interval *j*  (*j* = 1, 2, 3,…, 10); DD_*k*_ is the fixed effect of classes of days dry *k*  (*k* = 1, 2, 3, …, 10); b_1_AC⁡ and b_2_AC⁡ are the linear and quadratic regression coefficients, respectively, on age at calving in months; a_*l*_ is the random animal effect of cow *l*; c_*l*_ is the random permanent environmental effect of cow *l*; and e_*ijkl*_ is the random residual effects, e_*ijkl*_ ~ *N*(0, *σ*_*e*_^2^).

Model 2 was used for data sets 2 and 3. This model was similar to model 1. The only difference is that for data set 2 fixed effects of the previous calving interval and days dry were excluded because these values were just the same (zero) since cows had calved for the first time. The random effects of the permanent environmental effects were also excluded, since there were no repeat records within parity. For data set 3, the permanent environmental effect was excluded but the fixed effects of the previous calving interval and days dry were included in the mixed animal model. 

## 5. Results and Discussion

Variance components, heritability, and repeatability estimates are given in Table 4. 

Estimates of the additive genetic variances for yield traits in multiparous cows were dramatically smaller than those estimates from first lactation cows. As a result, heritability estimates for yield traits with repeated records were lower. The reason behind this is that in multiparous cows a repeatability animal model was used. One of the assumptions behind the use of repeatability models in the present study is that all lactations are genetically the same trait. This implies that the same set of genes exerts a common influence on the phenotypic expression of the first and later lactations [2]. This assumption is supported by observations that the genetic correlations among records are close to unity [10]. Conversely, changes in the additive genetic variances for the percentage traits in all records were small compared to those from the first lactation cows. Apparently, Jersey cows are able to systematically maintain milk composition throughout their lives [2]. 

The heritability and repeatability estimates for yield traits were somewhat lower than the average estimates of such estimates given by [11, 12], but heritability and repeatability estimates for protein and protein percentage are reported here for the first time for this breed. Banga [11] and Makuza et al. [12] reported heritability and repeatability estimates of 0.54, 0.56 and 0.36, 0.38 for milk yield in Zimbabwean Jersey cows, respectively. This is a little too high possibly due to method of estimation which was Harvey's least square mixed models by [11] and due to the small sample sizes by [12]. However, the results obtained in this study were slightly higher than those reported by [2] working with Jersey cattle population in a subtropical environment. Roman et al. [2] reported heritability estimates of 0.18, 0.14, and 0.10 for milk, fat, and protein yields, respectively. Differences in the estimates are expected as a result of differences in populations, in estimation methods, and in the mathematical models employed and because of sampling errors [13]. Small heritabilities (less than 0.10) mean that regardless of genetic evaluations and selection methods used, the genetic gain would be relatively small [12].

The heritability estimates for all records for SCC obtained in this study were not significantly different from zero. However, heritability estimates for SCC were lower in the first lactation and increases the later lactations, but they were also not significantly different from zero. This suggests that little genetic progress can be made by selection of this trait. High values of SCC are highly associated with mastitis [14]; therefore, the low heritability value (*h* ^2^ = 0.08) of SCC implies that substantial improvement has been made of Jersey herds in Zimbabwe from mastitis. As a result, this has led to good quality milk. 

The heritability estimates for all production traits are highest during the first lactation and decreases towards the later lactations. This is in agreement with the results reported by various researchers [2, 15, 16]. This result suggests a sizeable genetic maternal effect in the first lactation which drops with the second lactation and disappears by the third lactation [17]. 

The phenotypic and genetic correlations of 305-day production and SCC variables are given in Table 5. The genetic correlations of milk with yields of protein and fat and of protein yield with protein were very high, especially for milk yield with protein yield. Phenotypic correlations were higher than the corresponding genetic correlations. This means that an increase in protein yield can be achieved through selection of milk or fat yields. The correlation between protein yield and protein percentage in this study indicates that it was negative, but in general, this correlation is positive [18]. The cause for this is the high positive correlation between protein yield and milk yield and the high negative correlation between protein percentage and milk yield. Similar trend was also observed in the first lactation and later lactations. However, the correlations were generally high in the first two lactations than in later lactations and all records. The reason behind this is the low heritability estimates in later lactations and all records [19]. 

This study also shows that the genetic and phenotypic correlations between production traits and SCC were weak almost nonsignificantly, different from zero. Juozaitiene et al. [20] and Němcová et al. [21] also reported very weak genetic and phenotypic correlations, while [22, 23] reported absence of genetic correlation between production traits and SCC.

## 6. Conclusion

Variance due to permanent environmental effects was an important source of variation. The milk traits for Jersey dairy cattle in Zimbabwe were found to be more heritable than the SCC, and the correlation among the milk traits was significantly high. The correlation between the production traits and SCC was weak almost nonsignificantly different from zero. Therefore, these production traits may be preferred by breeders as selection criteria for development of effective genetic improvement programme. 

## Figures and Tables

**Table 1:** Restriction on age at calving by lactation.

Lactation	Age at calving (month)	
	Minimum	Maximum
1	22	36
2	34	48
3+	46	126

**Table 2:** Characteristics of the data sets.

	Parity			
	All	1	2	3+
Records	6725	1057	1117	4551
Sires	135	133	135	132
Dams	1093	1005	1054	1001
Herds	21	21	21	21
Paternal family	175			
Maternal family	14			

Means and standard deviations in parenthesis				
Milk (kg)	4468 (533)	4420 (527)	4528 (528)	4464 (535)
Fat (kg)	199 (36)	194 (35)	223 (41)	194 (33)
Protein (kg)	158 (25)	155 (25)	159 (25)	158 (26)
Fat%	4.42 (0.37)	4.35 (0.28)	4.89 (0.39)	4.33 (0.30)
Protein%	3.52 (0.15)	3.50 (0.16)	3.50 (0.14)	3.53 (0.15)
Log_10_SCC	5.54 (0.56)	5.32 (0.57)	5.38 (0.59)	5.50 (0.52)

**Table 3:** Pedigree structure means by Tier.

Tier	1	2	3	4
Individuals	6.00	277.00	64 43.00	11 60.00
Milk (kg)	—	44 64.84	45 39.21	45 38.83
Fat (kg)	—	198.57	205.45	211.13
Protein (kg)	—	157.78	160.88	161.53
Fat%	—	4.42	4.49	4.63
Protein%	—	3.52	3.53	3.54
Log_10_SCC	—	5.45	5.34	4.91

Tier 1: grand-grand-grandparents.

Tier 2: grand-grandparents.

Tier 3: grandparents.

Tier 4: parents.

**Table 4:** Variance components and genetic parameters (±s.e) for production and Log_10_SCC traits by parity.

Parity	Trait	*σ*_*a*_^2^	*σ*_pe_^2^	*σ*_*e*_^2^	*h* ^2^	*c* ^2^	*r*
All	Milk (kg)	86263	25532	173158	0.30 ± 0.10	0.09 ± 0.10	0.39
	Fat (kg)	428	78	822	0.32 ± 0.10	0.06 ± 0.10	0.38
	Protein (kg)	201	36	371	0.33 ± 0.10	0.06 ± 0.10	0.39
	Fat%	0.0587	0.0095	0.0710	0.42 ± 0.11	0.07 ± 0.11	0.49
	Protein%	0.0105	0.0016	0.0117	0.44 ± 0.12	0.07 ± 0.12	0.51
	Log_10_SCC	0.0252	0.0224	0.2500	0.08 ± 0.07	0.08 ± 0.07	0.16

1	Milk (kg)	106632	—	172145	0.38 ± 0.10	—	—
	Fat (kg)	482	—	750	0.39 ± 0.10	—	—
	Protein (kg)	247	—	386	0.39 ± 0.10	—	—
	Fat%	0.0364	—	0.0405	0.47 ± 0.12	—	—
	Protein%	0.0122	—	0.0126	0.49 ± 0.12	—	—
	Log_10_SCC	0.0357	—	0.2844	0.11 ± 0.07	—	—

2	Milk (kg)	73587	—	205573	0.26 ± 0.09	—	—
	Fat (kg)	533	—	1122	0.32 ± 0.09	—	—
	Protein (kg)	207	—	399	0.34 ± 0.10	—	—
	Fat%	0.0614	—	0.0910	0.40 ± 0.11	—	—
	Protein%	0.0087	—	0.0122	0.41 ± 0.11	—	—
	Log_10_SCC	0.0473	—	0.3039	0.13 ± 0.07	—	—

3+	Milk (kg)	61399	18852	206258	0.21 ± 0.08	0.07 ± 0.08	0.28
	Fat (kg)	245	77	779	0.22 ± 0.09	0.07 ± 0.09	0.29
	Protein (kg)	139	53	409	0.23 ± 0.09	0.09 ± 0.09	0.32
	Fat%	0.0282	0.0081	0.0515	0.32 ± 0.10	0.09 ± 0.10	0.41
	Protein%	0.0081	0.0023	0.0136	0.34 ± 0.10	0.09 ± 0.10	0.43
	Log_10_SCC	0.0420	0.0350	0.1952	0.15 ± 0.08	0.13 ± 0.08	0.28

*σ*_*a*_^2^: additive genetic variance, *σ*_pe_^2^: permanent environmental variance, *σ*_*e*_^2^: residual variance, *h* ^2^: heritability, *c* ^2^: permanent environment, *r*: repeatability, and SCC: somatic cell count.

**Table 5:** Genetic (below diagonal) and phenotypic (above diagonal) correlations and their standard errors between production traits and Log_10_SCC across and within lactations.

Parity	Trait	Milk (kg)	Fat (kg)	Protein (kg)	Fat%	Protein%	Log_10_SCC
	Milk (kg)		0.93 (0.00)	0.98 (0.00)	0.62 (0.21)	−0.78 (0.02)	−0.04 (0.03)
	Fat (kg)	0.91 (0.00)		0.93 (0.00)	0.86 (0.01)	−0.78 (0.02)	−0.01 (0.03)
All	Protein (kg)	0.95 (0.00)	0.91 (0.00)		0.64 (0.21)	−0.88 (0.01)	−0.03 (0.03)
	Fat%	−0.60 (0.21)	0.83 (0.01)	−0.61 (0.21)		0.59 (0.30)	−0.04 (0.03)
	Protein%	−0.75 (0.02)	−0.74 (0.02)	−0.86 (0.01)	0.55 (0.30)		−0.01 (0.03)
	Log_10_SCC	−0.03 (0.03)	−0.03 (0.03)	−0.01 (0.03)	−0.03 (0.03)	−0.01 (0.03)	

	Milk (kg)		0.98 (0.00)	0.98 (0.00)	0.95 (0.00)	−0.89 (0.01)	−0.01 (0.04)
	Fat (kg)	0.96 (0.00)		0.98 (0.00)	0.98 (0.00)	−0.94 (0.00)	−0.01 (0.04)
1	Protein (kg)	0.96 (0.00)	0.92 (0.00)		0.98 (0.00)	−0.95 (0.00)	−0.01 (0.04)
	Fat%	−0.94 (0.00)	0.95 (0.00)	−0.94 (0.00)		0.98 (0.00)	−0.02 (0.04)
	Protein%	−0.85 (0.01)	−0.89 (0.01)	−0.92 (0.00)	0.97 (0.00)		−0.01 (0.04)
	Log_10_SCC	−0.38 (0.32)	−0.59 (0.31)	−0.42 (0.32)	−0.63 (0.37)	−0.44 (0.27)	

	Milk (kg)		0.98 (0.00)	0.98 (0.00)	0.85 (0.01)	−0.89 (0.01)	−0.01 (0.04)
	Fat (kg)	0.92 (0.00)		0.97 (0.00)	0.93 (0.00)	−0.87 (0.01)	−0.01 (0.04)
2	Protein (kg)	0.92 (0.00)	0.97 (0.00)		0.83 (0.01)	−0.94 (0.00)	−0.01 (0.04)
	Fat%	−0.85 (0.01)	0.89 (0.01)	−0.76 (0.02)		0.75 (0.02)	−0.05 (0.07)
	Protein%	−0.87 (0.01)	−0.89 (0.01)	−0.88 (0.01)	0.72 (0.02)		−0.01 (0.04)
	Log_10_SCC	−0.49 (0.36)	−0.63 (0.42)	−0.57 (0.32)	−0.69 (0.37)	−0.56 (0.32)	

	Milk (kg)		0.95 (0.00)	0.98 (0.00)	0.62 (0.21)	−0.75 (0.02)	−0.05 (0.07)
	Fat (kg)	0.92 (0.00)		0.97 (0.00)	0.82 (0.01)	−0.81 (0.01)	−0.04 (0.07)
3+	Protein (kg)	0.96 (0.00)	0.92 (0.00)		0.68 (0.21)	−0.85 (0.01)	−0.04 (0.07)
	Fat%	−0.59 (0.32)	0.78 (0.02)	−0.63 (0.21)		0.73 (0.02)	−0.01 (0.04)
	Protein%	−0.72 (0.02)	−0.78 (0.02)	−0.81 (0.01)	0.68 (0.21)		−0.01 (0.04)
	Log_10_SCC	−0.19 (0.04)	−0.18 (0.04)	−0.18 (0.04)	−0.14 (0.04)	−0.13 (0.04)	
